# Supplementary material for: Generation of Iron-Independent Siderophore-Producing Agaricus bisporus through the Constitutive Expression of hapX
Source: Genes (Basel). 2021 May 13;12(5):724. doi: 10.3390/genes12050724 (PMC8152254; doi:10.3390/genes12050724)
Supplement: Supplementary file 1 [file genes-12-00724-s001.zip › genes-1191937-supplementary/genes-1191937-suppl/Supplementary Table S1.pdf]

genes

MDPI

Supplementary Table S1. Primers used in this study.

	Primer sets	Sequence(5' to 3')
Cloning of *hapX*	hapX-F	GAATTCATTTAAATCATGTCCGCAGCACTCCTAC
	hapX-R	GAATTCGGGCCCATTTGGGGAACGTCAGTCGT
Screening of transformants	hph-F	CAGCGTCTCCGACCTGATG
	hph-R	GAAATCCGCGTGCACGAGG
	Pgpd-F	CGAGGCTTGCGTCATTCTGTG
	hapXin-R	TAACCCCCTTTCTCGAGAAC
DIG-labeled *hapX* probe	hapX-probe-fwd	GACGCAAGATTGAACGACCG
	hapX-probe-rev	CCTCTCAGTTCCGAATGGTAG
qPCR analysis	hapX-rt-F	GGCCGATCTTGAAATGGAGTC
	hapX-rt-R	FATTGAACGACCGGGGAAGC
	sidA-rt-F	CTATGTATTCCCCACTCAAGG
	sidA-rt-R	TTTTCGGGTCGACAAGAGCC
	sidC/E-rt-F	CGGAAACGTCGTTTCAGACAG
	sidC/E-rt-R	GCCATGTGAAGATTTCCCGGA
	sidD-rt-F	CAGCTCAAGCAACGCGTCAA
	sidD-rt-R	ATAGATTTCGCCGACCTCGC
	β-tubulin-rt-F	GGTGCTGGTATGGGTACCC
	β -tubulin-rt-R	TTGACCGGGGAAGCGCAG
