# Supplementary figures and images for: Generation of Iron-Independent Siderophore-Producing Agaricus bisporus through the Constitutive Expression of hapX
Source: Genes (Basel). 2021 May 13;12(5):724. doi: 10.3390/genes12050724 (PMC8152254; doi:10.3390/genes12050724)

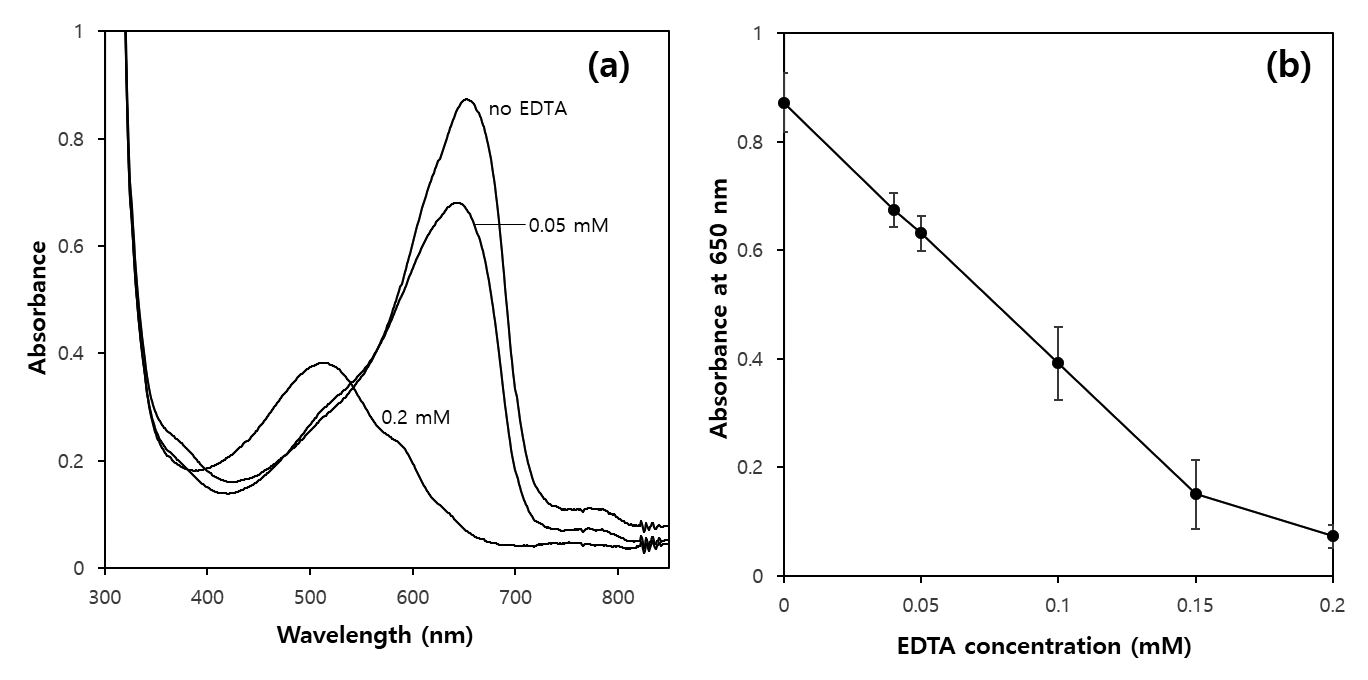

Supplement: Supplementary file 1 [file genes-12-00724-s001.zip › genes-1191937-supplementary/genes-1191937-suppl/Supplementary Figure S4.TIF]

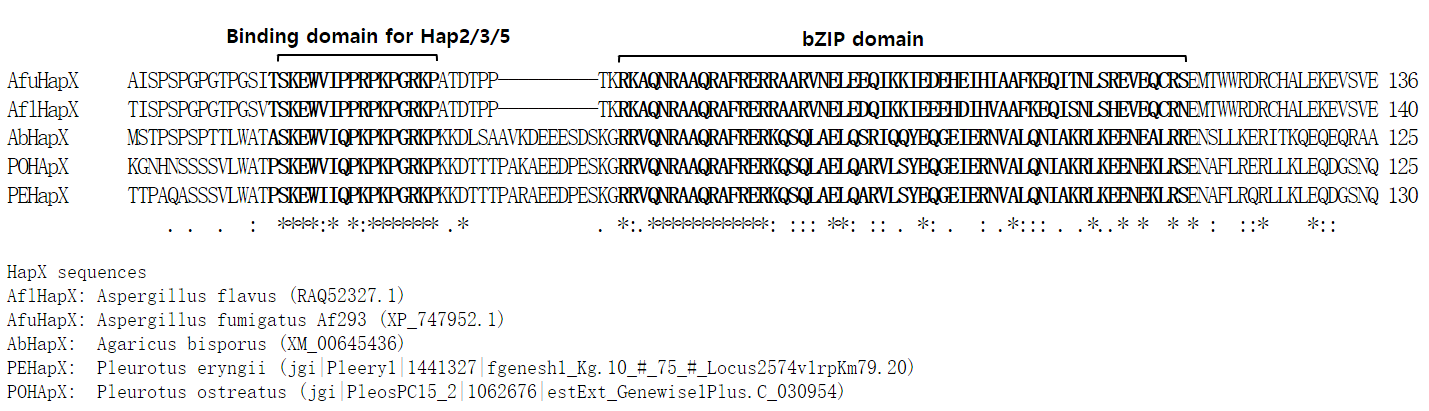

Supplement: Supplementary file 1 [file genes-12-00724-s001.zip › genes-1191937-supplementary/genes-1191937-suppl/Supplementary Figure S1.tif]

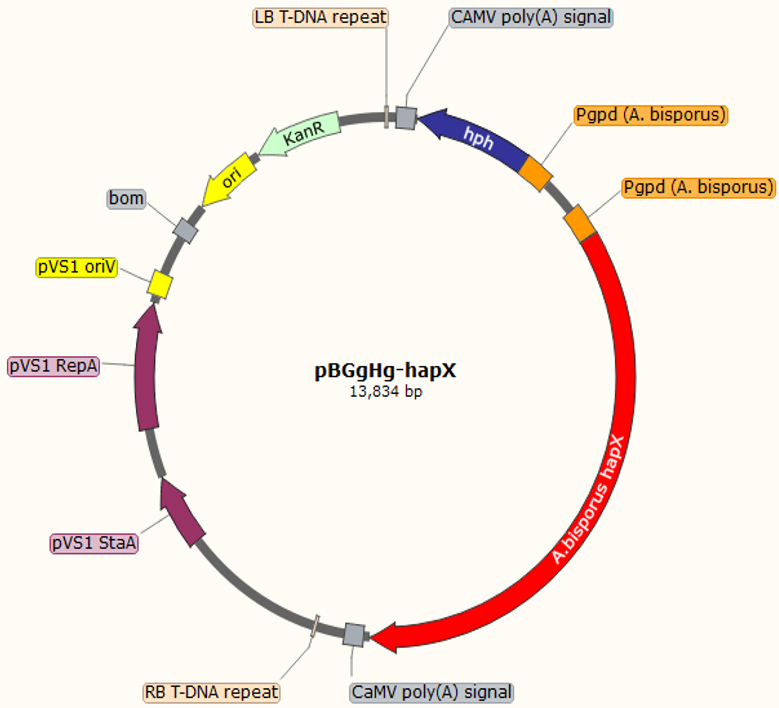

Supplement: Supplementary file 1 [file genes-12-00724-s001.zip › genes-1191937-supplementary/genes-1191937-suppl/Supplementary Figure S2.tif]

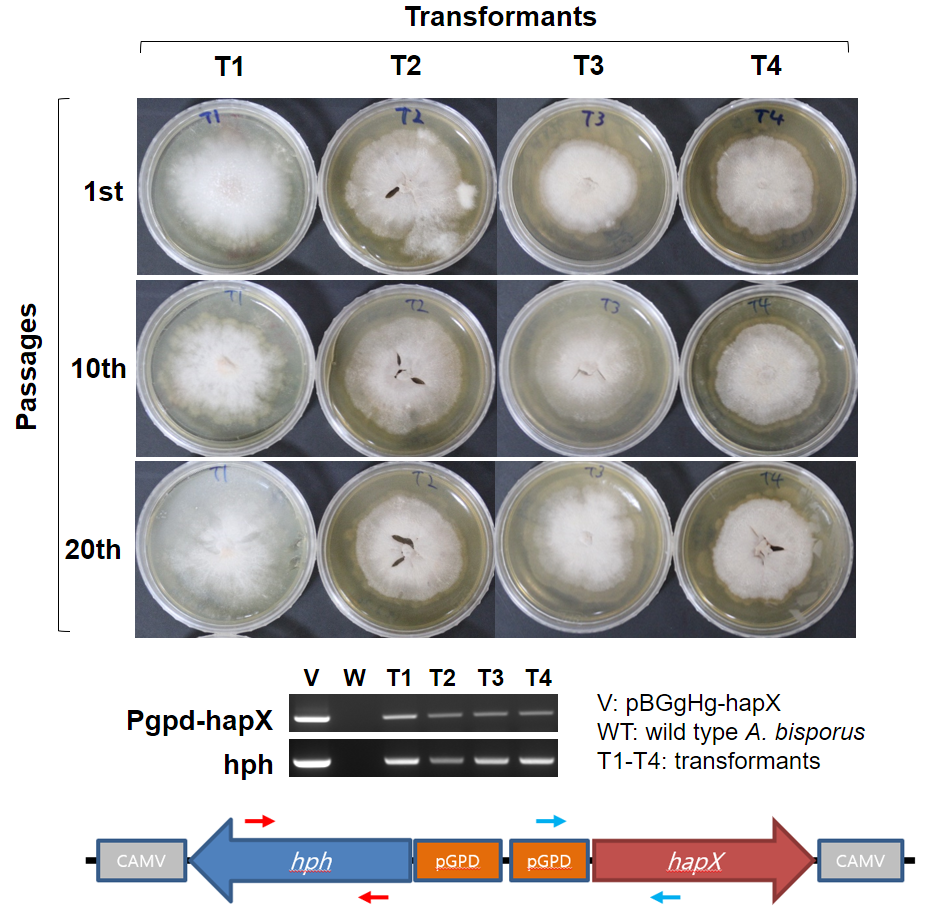

Supplement: Supplementary file 1 [file genes-12-00724-s001.zip › genes-1191937-supplementary/genes-1191937-suppl/Supplementary Figure S3.TIF]
